# Professional Courtesy

**Published:** 1875-09

**Authors:** W. C. Sheppard

**Affiliations:** Columbia, Tenn.


### PROFESSIONAL COURTESY

BY W. C. SHEPPARD, COLUMBIA, TENN.

*Read before the Tennessee State Dental Society.*

In all the learned professions, and indeed among all men who pursue the same vocation, there are certain rules and regulations, touching the conduct of one to another, which are obtained by mutual consent.

The members of the legal profession, have rules which regulate their conduct, both with the public, their clients and with each other; which if wantonly and ruthlessly disregarded do not fail to bring the offender into shame and disgrace with his brethren. In a high toned and intelligent community, the lawyer who underbids his fellows; depreciates openly their ability, or business qualities, or fails to treat even the youngest member of the profession with kindness and courtesy, is sure to fall into disrepute, and finally to be frowned down by all. In the practice of law. it is not an unusual thing for a young practitioner, before taking steps in a suit, to consult some one of the older members and get his advice as to the law of the case and mode of proceeding.

And this would not be refused, even though the older member had reasons to believe that he himself would probably be employed on the other side. The medical profession not less than the legal, has also its rules of etiquette and courtesy, and it is not going too far to say that no physician dare, with impunity, to violate the well known and established rules by which the profession is governed, in its intercourse the one with the other. In both legal and medical profession it is one of the highest marks of professional courtesy, that the oldest and best informed are always solicitous, to aid the younger members, in their honest efforts, to arrive at professional skill and success. If I am not misinformed as to these things; the old lawyer or physician who would refuse to give to his young and ambitious brother, the benefits of his superior learning and experience, on all proper occasions, would be regarded by the profession, as a disgrace to its ranks.

All this of course, does not by any means preclude the idea, that the honorable portion of either profession, would not be slow to condemn in the most unmistakable terms, the knave, the shyster, the quack and the charlatan.

The remarks I have made, are only applicable to those who pursue their respective professions in an honorable line, and who have some other ambition save the mere question of gain. Our profession, in some respects, is in its infancy. True! wonderful progress has been made towards perfection, and now more particularly, in the last few years, and we may with almost certainty, look for rapid progress in the future. In the last few years, its advancement has been such, that it now ranks, as one of the learned professions, and contains within its circle, some of the most learned and useful men of the country. Its benefits, and blessings upon the community at large, are felt and acknowledged everywhere. And those who are reallv worthy, are reaping as much reward, for their learing and skill, as the members of any other profession. Standing thus, before the world, is it not due to ourselves and to our vocation, that we should be governed in our conduct towards the world, and especially towards each other, by rules and principles, which will add, not only to our pleasure, but which will be sure to elevate and dignify; the station of professional exellency? It is said that we have many ignorant and unworthy members in our profession. If this be true, why is it so? If we would deter the ignorant, and unworthy from entering our ranks, we should so elevate and dignify the profession, that the *low*, *vulgar* and *unprincipled*, can not stand the light of an enlightened and gentlemanly profession. We should ever forbear to comment severely on the young, but earnest and honest beginner in the profession. Rather let us aid and encourage him, let us instruct him, and should he inadvertently make a mistake, let us go to him first, and give him an opportunity to correct his error.

How shameful it is, to hear a member of the profession, by hints, inuendoes and insinuations, seeking to bring into contempt, a young professional brother. If we are called upon to operate on a patient, who has had the services of some other dentist, and should discover what we consider to be an error in his work, would it not be better and far more dignified and gentlemanly, to repair the work and make no comments? If our own operation is scientific and successful, the patient will be sure to find it out himself, and will not be slow to let it be known to his friends, and thus save us the trouble of blowing our own trumpets at the expense and mortification of a fellow laborer in the profession. It has fallen to the lot of the writer, whilst honestly struggling to win an honorable position in his profession, to be a sufferer from unprofessional conduct, like that to which we have refered, and had it not been for a dogged determination on his part to allow no difficulties to discourage him, he might have long ago, retired from the ranks of the profession in disgust.

We trust it will not be considered immodest on the part of the writer, to say this much.

All men have not sufficient courage to stand the assaults of envy, malice and detraction, and we doubt not that many a brilliant and noble spirit has been banished from the profession by reason of unnecessary and wanton attacks, upon their professional character, made by men who were themselves unworthy.

Another means of depreciating the character of the profession, is that of underbiding for practice, though we are glad to know, that this does not now exist to the extent that it did formerly, yet we are by no means free from it.

As a general rule, it is not only the right, but it is the duty, of every person to obtain professional services, at the lowest rates they can, and while we are sincerely opposed to extortion or professional combination, to run up prices, we are as earnest in our advocacy of sound living prices for our labor, and he, who for the sake of building up a practice, charges less than usual, and living rates, is wholy unworthy of the profession.

Our profession is one which is taking a high stand in the country.

Its rewards, (though sometimes delayed,) are sure to come to him who cultivates the virtues of industry, patience, investigation and honorable ambition. It is justly regarded as an indispensable profession to the world, and whilst we are struggling to enlarge the sphere of its usefulness, let us not forget to elevate its standard of morals, ennoble and dignify, the characters of its members, so that to be a scientific, successful dentist, will not only insure gain, but be a passport to the best and most enlightened circles of society.

